# Case report: The theory of post-ileocystoplasty spherical configuration in patients with low-capacity bladder

**DOI:** 10.1016/j.ijscr.2021.105731

**Published:** 2021-03-05

**Authors:** Dwiki Haryo Indrawan, Yacobda Sigumonrong

**Affiliations:** a Department of Urology, Faculty of Medicine, Universitas Indonesia – Haji Adam Malik General Hospital Medan, Jl. Bunga Lau No.17, Kemenangan Tani, Kec. Medan Tuntungan, Kota Medan, Sumatera Utara, 20136, Indonesia; b Division of Urology, Department of Surgery, Faculty of Medicine, Universitas Sumatera Utara – Haji Adam Malik General Hospital Medan, Jl. Bunga Lau No.17, Kemenangan Tani, Kec. Medan Tuntungan, Kota Medan, Sumatera Utara, 20136, Indonesia

**Keywords:** Bladder augmentation, Ileocystoplasty, Detubularization, Case report

## Abstract

**Introduction:**

Bladder augmentation can be performed by detubularization of the small or large intestine. A large capacity bladder is necessary to avoid frequent urination; thus, the ileal sac must be able to store the maximum volume of urine with relatively low pressure and the shortest length of the intestine. The acceptable pressure capacity should always be under ureteric pressure to avoid back pressure and kidney damage. Large capacity at low pressure is referred to as good compliance. Desirable preparations for augmentation, which is the use of the shortest length of the intestine, reduce the chance of diarrhea and vitamin deficiency and retain the intestines which may be required for augmentation.

**Aim:**

Clinical and urodynamic evaluation of the recent postoperative condition of the patient who underwent ileocystoplasty, confirmed by the theory of detubularization (spherical) configuration.

**Case presentation:**

Patient with complaints of frequent urination and small amount of urine. Ultrasound examination showed low volume bladder capacity and bilateral hydronephrosis and hydroureter. From cystography and VCUG examination, low capacity bladder, grade 1 VUR on the right side, grade 4 VUR on the left side accompanied by bilateral hydronephrosis and hydroureter. The patient has a history of right nephrectomy in 2014 for pyonephrosis due to kidney stones. The patient was then subjected to bladder augmentation using a segment of the ileum (ileocystoplasty) in 2015. In the postoperative evaluation, clinical symptoms, radiological and uroflowmetric examinations were evaluated.

**Conclusion:**

The detubularization form offers greater volume and lower pressure in the reservoir to augment the bladder.

## Highlights

• Detubularization form offers greater volume and lower pressure in the reservoir to augment the bladder.
• Patients with low-capacity bladder who have poor compliance with ureteral involvement have poor renal function.
• Low compliance bladder will cause numerous symptom such which will lead to insufficient bladder emptying.
• Bladder augmentation is a management option for neurogenic and non-neurogenic bladder dysfunction when conservative management, medical pharmacological therapy and minimally invasive management have not yielded satisfactory results.
• The aim of doing bladder augmentation is to create a reservoir with adequate functional capacity and low bladder filling pressure so that low intravesical pressure will not interfere with the flow of urine from the bladder to the urethra.

## 1 Introduction

Bladder augmentation is a classic and effective surgical procedure for the management of patients with small capacity bladder that has the ability to collect small amount of urine with or without overactivity of the detrusor muscle [1]. The main indication of this procedure are cases of neurogenic or non-neurogenic bladder dysfunction that did not respond to medical therapy, congenital disorders of the bladder, and infection or inflammation of the bladder. Bladder augmentation can be performed using an anastomosed intestinal segment into the bladder so that the size of the bladder will increase and prevent the reflux of urine into the upper urinary tract [2]. In 1899, Mikulicz reported the bladder augmentation using one part of the small intestine for the first time. This case report discussed the bladder augmentation performed on a 13-year-old boy with small bladder capacity at RSUP Haji Adam Malik Medan. This case report was made according to the SCARE guideline [3].

## 2 Case report

A 13-year-old boy came to RSUP Haji Adam Malik Medan on March 20, 2014 complaining of frequent urination and only 40–60 cc of urine came out every time he urinates. This complaint has been experienced by the patient since the patient was 8 years old. The patient complained of urinating at intervals of every 30 min every urination. Intermittent back pain on the right side was experienced by the patient one year ago. On ultrasound and BNO-IVP (Blass Nier Overzicht – Intravenous Pyelography) examination, non-visual conclusion, right kidney pyonephrosis and left kidney hydronephrosis were found. The patient then underwent a right kidney nephrostomy on March 24, 2014 due to pyonephrosis. From the CT scan of the abdomen on March 25, 2015, multiple stones of the right kidney were found, accompanied by bilateral hydronephrosis and hydroureter. Then a nephrectomy was performed on April 24, 2015 for pyonephrosis and non-visual right kidney. On April 7, 2015 the patient underwent cystography and voiding cystourethrogram (VCUG) with the conclusion of low-capacity bladder, grade 1 vesicoureteral reflux (VUR) on the right side, grade 4 VUR on the left side accompanied by hydronephrosis and bilateral hydroureter. In addition, the patient was diagnosed with neurogenic bladder after that. The patient was then subjected to bladder augmentation due to low-capacity bladder in September 2015. The patient in supine position, under general anesthesia, underwent a lower midline abdominal incision. After the bladder was identified, a vertical incision was made from the anterior bladder to the posterior bladder. The ileum was identified and then 20 cm long ileal segment was taken and an end-to-end anastomosis was performed. The ileal segments are then washed with normal saline and betadine solutions. Afterwards, the ileal segment is incised and is reconstructed to form a dome. The part of the ileum that had been reconstructed was anastomosed to the bladder using Monocryl 3.0 then a cystostomy was placed. The patient then underwent 2 weeks postoperative care with normal urine production and cystostomy. In the first month of postoperative follow-up, the symptom frequency was reduced to 25–30 times per day. From the pre- and post-micturition ultrasound evaluation first month after bladder augmentation, the pre-micturition bladder volume was 221 cc and the post-micturition bladder volume was 70 cc. In the second- and third-month visits, the patient conveyed reduced frequency symptoms, urinate for 15–25 times per day, and from the ultrasound evaluation, the pre-micturition bladder volume was 350 cc and the post-micturition bladder volume was 50 cc. The patient then did not come back but came back for visit in the third year. From the interview, it was found that the frequency symptoms in the first, second and third year was 6–8 times micturition per day, and at the end of the micturition the patient had to change positions and press the supra-symphysis area until it felt light. In the third year, the patient came for visit and was followed-up for clinical symptom, ultrasound, cystoscopy, VCUG and urodynamics, all of which showed good results. From VCUG examination on June 28, 2019, bladder fullness sensation was found on 250 cc bladder filling, no reflux, open bladder neck, and good sphincter. The patient had to change sitting and standing positions while pressing on the supra-symphysis area, with a urine residue of 40 cc. Cystoscopy performed on the same date showed good bladder mucosa, good ileal augmentation anastomosis to the bladder, and ureteral estuary can be identified with a bladder capacity of 350 cc (Table 1).

**Table 1:** Comparison of Symptoms and Frequency before and after surgery.

Voiding complaint	Frequency symptom
Before surgery	60–80 times a day
1 month after surgery	25–30 times a day
2 months after surgery	20–25 times a day
3 months after surgery	15–20 times a day
2 years after surgery	6–8 times a day
3 years after surgery	6–8 times a day

The patient was then subjected to uroflowmetry twice on June 30, 2018, with the results of 20 s urination time, 18.8 s flow time, total urine of 544.8 cc, average flow rate of 29.1 mL / second, 41.3 s maximal flow, and 7.5 s time to maximum flow.

## 3 Discussion

Pathological infections, such as genitourinary tuberculosis, schistosomiasis, and post-radiotherapy cystitis are often the cause of low-capacity bladder cases with or without ureteral involvement [2,4]. Patients with low-capacity bladder who have poor compliance with ureteral involvement have poor renal function. Bladder compliance is a concept where the bladder should retain low intravesical pressure while the volume increasing to a certain point. It is a measure that every urodynamic care provider should calculate. Low compliance bladder will cause numerous symptom such which will lead to insufficient bladder emptying. On the long run, this issue could end as Chronic Kidney Disease and increase patient mortality. Therefore, these patients need proper management [5].

Most patients with bladder dysfunction problems can be managed medically without surgery. Treatment before surgery can include the use of antispasmodics and intermittent catheterization [[5], [6], [7]]. Bladder augmentation is a management option for neurogenic and non-neurogenic bladder dysfunction when conservative management, medical pharmacological therapy and minimally invasive management have not yielded satisfactory results. If these therapies are unsuccessful, patients who fail with these therapeutic modalities can be recommended for bladder augmentation [7,8] The aim of doing bladder augmentation is to create a reservoir with adequate functional capacity and low bladder filling pressure so that low intravesical pressure will not interfere with the flow of urine from the bladder to the urethra. It also prevents high pressure of upper urinary tract that can lead to vesicoureteral reflux (VUR). In this patient, the indication for bladder augmentation is low-capacity bladder which does not improve with medical therapy.

**Fig. 1:**
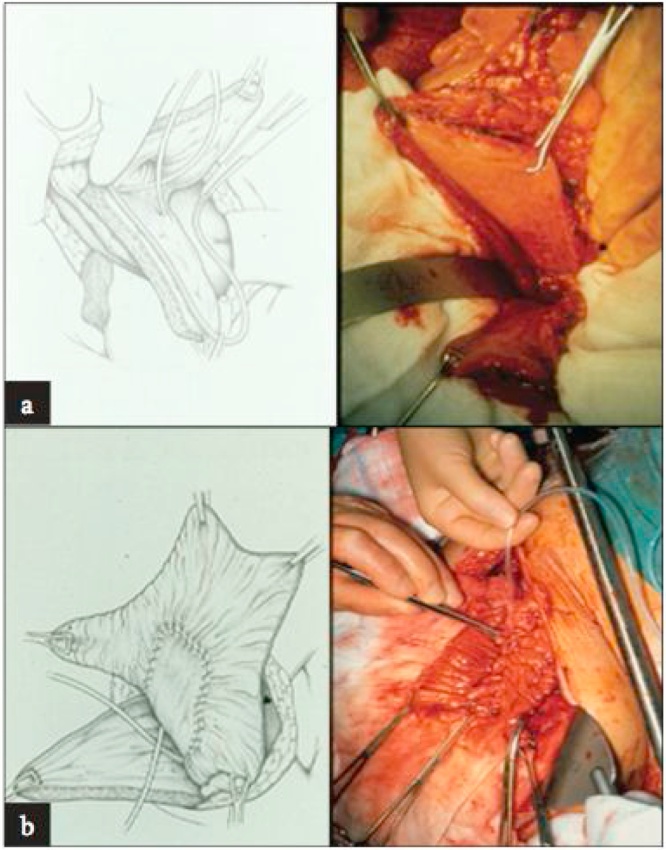
(a) the bladder that has been split (b) anastomosis of ileum and bladder.

Various types of gastrointestinal segments have been used for bladder augmentation, but the ileum is the most frequently performed segment [9,10]. Von Mikulicz describes ileocystoplasty augmentation in 1889 [11]. Hinmann and Koff also describe the benefits of opening the bowel at the antimesenteric border, detubularization, and reconfiguration [12]. McGuire demonstrates risk of increased intravesical pressure on renal function [13]. The spherical shape is the most desirable configuration because it has a maximal volume for the intended surface area, leading to dullness of bowel contractions and an overall increase and compliance according to Laplace's law [14] (Fig. 1).

The size and configuration of the augmentation segment may be more important than the type of intestine used. Hinman (1988) and Koff (1988) have clearly demonstrated the advantage of opening the intestinal segment at antimesenteric border, thus enabling detubularization and reconfiguration of this segment. Detubularization and reconfiguration maximizes the additional surface area to the bladder and thus benefits from a specific segment. Furthermore, intrinsic innervation is impaired and peristalsis is significantly reduced. Reconfiguration to a spherical shape provides many advantages that increase capacity and overall compliance. Spherical configuration with geometry, maximizes volume achieved for a given bladder wall area. In addition, the spherical configuration maximizes the radius of curvature, thereby increasing the surface tension for exerted bladder pressure, which tends to lead to further bladder expansion. This is a relationship of Laplace's law (T = k RP), where T is the wall stress, k is a constant depending on the elasticity and characteristics of the wall, R is the radius of curvature, and P is the luminal pressure [15].

**Fig. 2:**
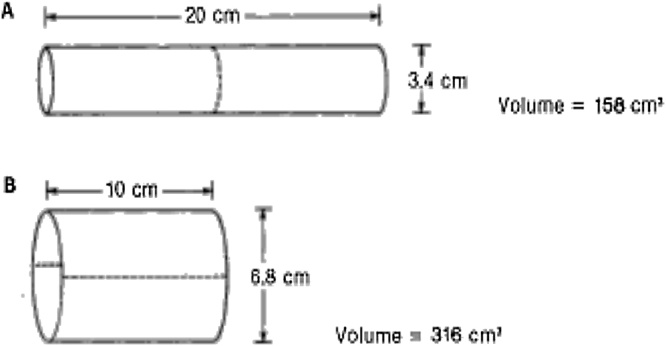
Comparison of the tube capacity calculation with a length of 20 cm and a diameter of 3.4 cm (175cm^2^) and on the same tube that is opened and folded lengthwise with a length of 10 cm and a diameter of 6.8 cm (316 cm^2^) [15].

**Fig. 3:**
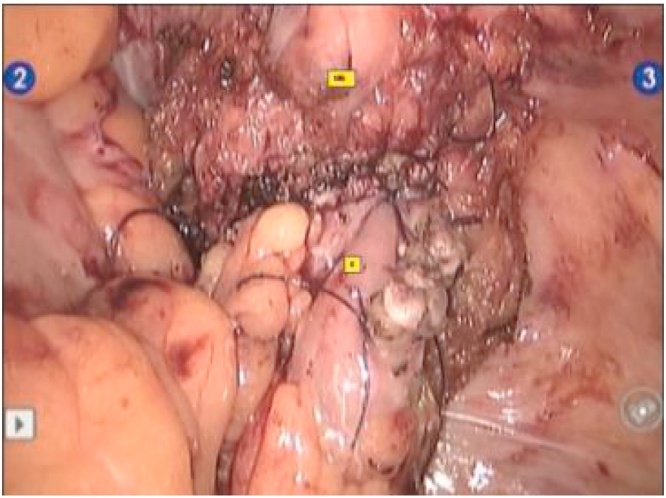
Anastomosed ileum and bladder.

The geometric effect can be seen in the following example: 20 cm long tube with a diameter of 3.4 cm (equivalent with physiological diameter of the ileum) had a calculated capacity of 175 mL (Fig. 2A). If opened longitudinally and folded back as a bag, the capacity calculated by the equation will be close to 350 mL, twice that of the tube (Fig. 2B) [12] (Fig. 3).

In this patient, bladder augmentation was performed by taking a segment of the ileum (ileocystoplasty) on indication of low-capacity bladder. Some urologists use the small bowel segment in bladder augmentation as the procedure of choice when conservative management fails. In 1982, Good win described this procedure, which was later popularized by Mundy and Stephenson. [16] Mundy et al. reported a 90% success rate in cystoplasty augmentation performed in 40 patients with neuropathic bladder dysfunction with a mean follow-up of one year. Several other reports have confirmed the high success rate of obtaining a high capacity and low-pressure reservoir.

Krishna et al. studied 39 children with spina bifida and reported a 91.7% reduction in upper urinary tract dilatation. [17] Riedmiller et al., in a case report used the ileal segment for a bladder augmentation procedure. Post-treatment follow-up showed increased bladder compliance with moderate mucus production. The complication rate with this technique is reportedly low [18].

In the literature it is stated that the ileal segment required for this procedure is at least 20 cm and is located next to the proximal ileocecal valve [19,20]. This is consistent with the technique used in this case report. In a case report by Ahmad El-Feel et al., in 2008 on 23 patients with an age range of 12–56 years and an average age of 27 years, it was stated that the use of 10–15 cm ileal segments could increase the bladder capacity from an average of 111 ml–788 ml at 1 year postoperatively and also decreased the detrusor pressure from a mean of 92 cmH2O to 15 cm H2O [21].

In the statistical analysis of Schmidbauer et al., pressure volume curves showed significantly better compliance with detubularization than ileal segment tubularization. The area under the curve (AUC) p < 0.02 after 12 weeks was 21.2 vs 85.3 (cmH2O). [22] Goldwasser et al. stated that the mean bladder volume at maximal contraction was higher in patients with detubularization than with tubular post-ileocystoplasty. The maximal contraction that occurs at a lower bladder volume and a higher amplitude was more likely occur in patients with tubular ileocystoplasty [23].

Shadpour et al. reported a serial case of 6 patients with low-capacity bladder with myelomeningocele which had undergone laparoscopic augmentation ileocystoplasty in combination with Malone procedure (using appendix segment as a conduit). The result was that the mean bladder capacity before the procedure was 48 mL, and a mean of 260 mL in 13–16 months after treatment. Detrusor pressure was decreased, from a mean of 35 cm H2O to 12 cm H2O. Khastgir et al., in 2003 reported 32 patients, consisted of 25 male patients and 7 female patients with an age range of 11 years-52 years with a mean age of 32.6 years, who were augmented with the ileocystoplasty technique. The results showed that the mean capacity of the pre-micturition bladder from an average of 143 cc to a mean of 589 cc in one-year post-treatment evaluation and decreased detrusor pressure from a mean of 108 cmH2O to 19 cmH2O in post-treatment evaluation which conducted after a year. [24] Quek et al. in 2003 reported a serial case of 26 cases of enterocystoplaty in pediatric patients with neurogenic bladder who failed conservative therapy with a mean age of 8 years. The mean bladder capacity was 201 cc to 618 cc at the fourth-year post-procedure evaluation and a reduction in detrusor pressure from an average of 81cmH2O to 12 cmH2O [25]. In the case report of laparoscopic robotic ileocystoplasty augmentation by Kang, 2010, ileal resection was carried out along a 15 cm long segment of the ileum and formed a U-shaped ileal bag. The bladder function capacity increased to 280 cc and the residual volume of urine was 5 cc or less [26].

**Fig. 4:**
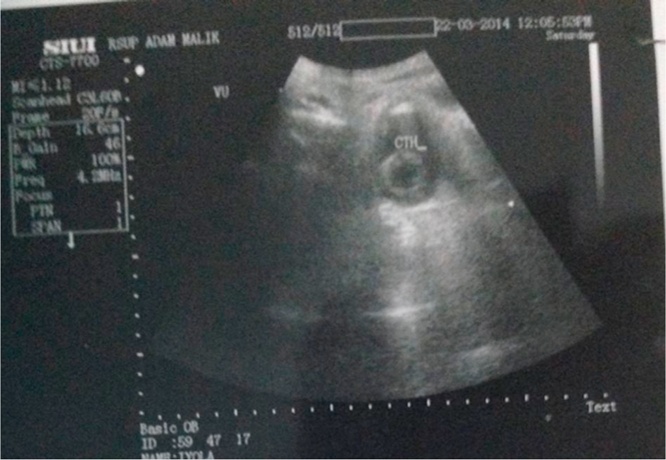
The patient’s ultrasound before bladder augmentation.

**Fig. 5:**
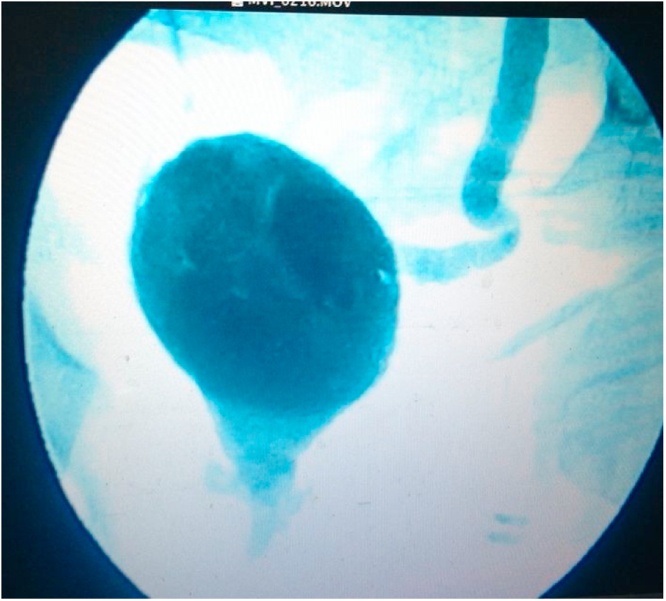
The patient’s VCUG (Voiding Cystouretrography) before bladder augmentation.

**Fig. 6:**
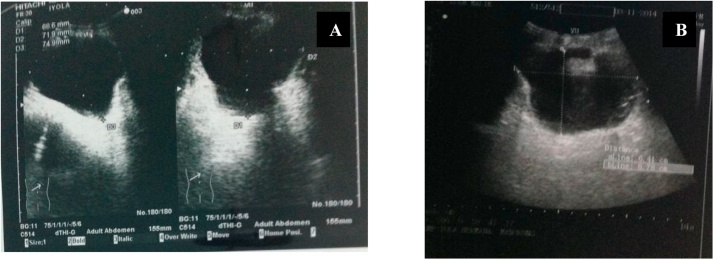
The patient’s post-bladder augmentation ultrasound (A. 1 month after surgery, B. 2 months after surgery.

**Fig. 7:**
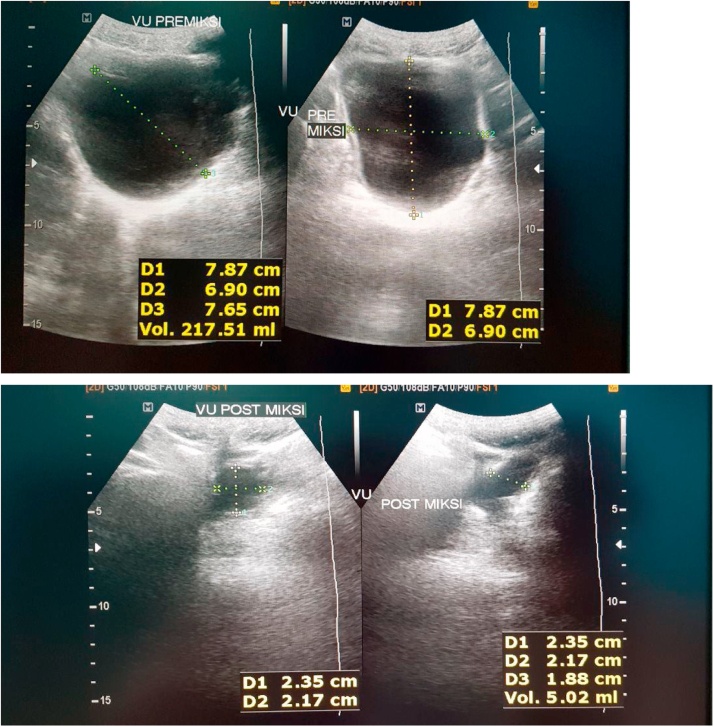
Pre-micturition and post-micturition ultrasound of post-bladder augmentation patient (3 years after surgery).

**Fig. 8:**
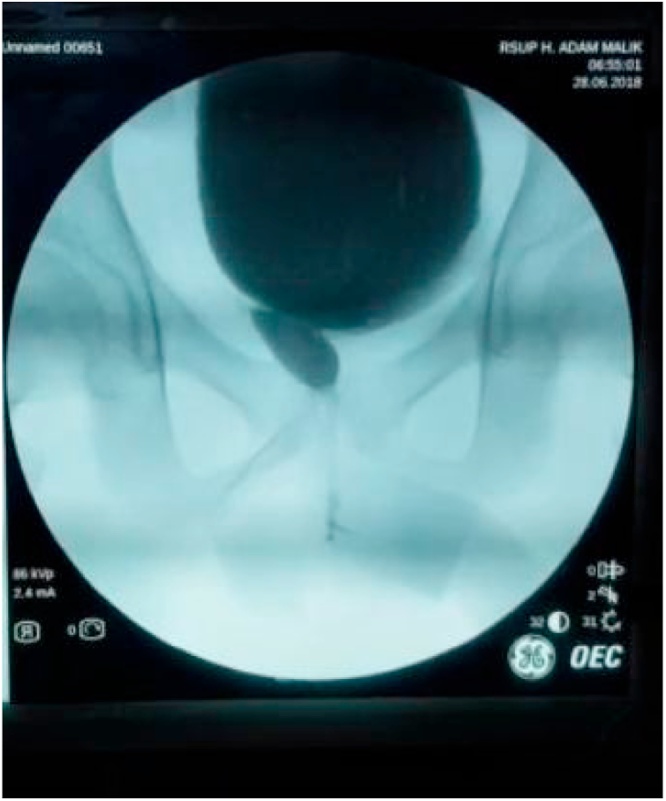
The patient’s VCUG in the third-year post bladder augmentation.

**Fig. 9:**
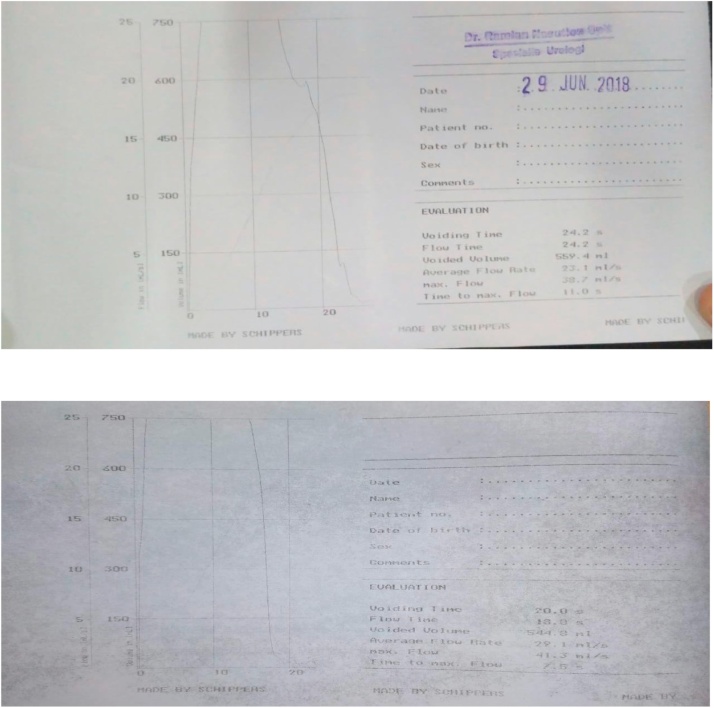
The patient’s urodynamic in the third-year post bladder augmentation.

In this study, the results of post augmentation bladder volume in the first month were 221 cc and post micturition bladder volume was 70 cc. After 3 months of monitoring, it was found that the volume of pre-micturition was 350 cc and the post-micturition was 50 cc. Three years after the bladder augmentation, our patient's bladder capacity increased to 545 cc. This is in accordance with La Place's theory that Hinnman and Koff did in 1988 where the bladder capacity is expected to increase maximally with the use of a shorter intestinal lumen. However, there is another theory where the bladder capacity in children continues to increase due to the growth and development of the bladder volume according to their age (Fig. 4, Fig. 5, Fig. 6, Fig. 7, Fig. 8, Fig. 9).

## 4 Conclusion

In this case, the bladder augmentation was performed on the indication of a low capacity bladder by using the ileocystoplasty technique, which is a technique that uses reconstructed ileal segment which anastomosed to the bladder. Bladder augmentation in this case requires good care because it can cause several complications such as infection, stone formation, malignancy, metabolic disorders, perforation, dysuria, hematuria and low back pain [[11], [12], [13]]. In the first month, second month, to third year evaluation after surgery, there was improvement of urinary symptoms, USG, VCUG and uroflowmetry. This is in accordance with the theory of ileal detubularization which offers a greater volume and lower pressure in the reservoir during bladder augmentation.

## Declaration of Competing Interest

The author(s) have no conflict of interest to declare.

## Funding

This case report source of funding is self-funding.

## Ethical approval

This case report has been exempted from ethical approval by Universitas Sumatera Utara Ethical Committee.

The patient dan his parents have given their consent in order for us to publish this case. We have informed the patient and his family comprehensively and they gave us their consent and fully supported the publication of this study.

## Author contribution

DHI carried out the data collection, analyzing the data and and drafted the manuscript. YS participated in the design of the study and helped to draft the manuscript. All authors have read and approved the manuscript.

## Registration of research studies

Not Applicable.

## Guarantor

Dwiki Haryo Indrawan M.D.

## Provenance and peer review

Not commissioned, externally peer-reviewed.
